# Triazole-Resistant *Aspergillus fumigatus* from Fungicide-Experienced Soils in Naivasha Subcounty and Nairobi County, Kenya

**DOI:** 10.1155/2018/7147938

**Published:** 2018-06-26

**Authors:** Edson K. Kemoi, Andrew Nyerere, Christine C. Bii

**Affiliations:** ^1^Jomo Kenyatta University of Agriculture and Technology, Nairobi, Kenya; ^2^University of Kabianga, Kericho, Kenya; ^3^Kenya Medical Research Institute, Nairobi, Kenya

## Abstract

The mainstay in prevention and treatment of aspergillosis is the use triazole drugs. In Kenya, the use of agricultural azole is one of the predisposing factors in development of resistance. One hundred fifty-six (156) experienced soils were collected from agricultural farms and cultured on Sabouraud DextroseAagar. The study isolated 48 yielded *Aspergillus fumigatus* and 2 *A. flavus*. All the isolates were subjected to antifungal susceptibility testing against three triazoles: posaconazole, voriconazole, and itraconazole. Out of the isolates, 3 had MIC of 32 and 1 had MIC of 16 against itraconazole, and 1 isolate had MIC of 32 against posaconazole. CYP51A gene was sequenced, and TR34/L98H mutation was identified. Triazole resistance existing in Kenya calls for rational use of azole-based fungicides in agriculture over concerns of emerging antifungal resistance in clinical practice.

## 1. Introduction

*Aspergillus* species, especially *Aspergillus fumigatus*, is the most common cause of aspergillosis, which is the second leading cause of death after cryptococcosis in patients suffering from fungal infections [1]. Azole-resistant *Aspergillus fumigatus* is an evolving global health challenge [2]. It is a frequent colonizer of cavitary lesions in tuberculosis patients and cause of mortality in post-TB treatment cases [3].

Aspergillosis treatment is done by using amphotericin B or azoles. However, resistance against azoles has been increasingly reported especially from high- and middle-income countries [4–12]. Previous studies from different regions of the world—African region (Tanzania), European region (Netherlands, Belgium, Denmark, and Germany), Asia (Kuwait, India, and Iran), and the USA—have reported multiple sources of azole-resistant *Aspergillus fumigatus* from soil sample, flower beds, plants, compost, and hospitals and its environs [4, 10, 12–23].

In contrast, limited data are available from low-income countries, especially from sub-Saharan Africa. However, a recent disturbing report of high resistance to azole was reported in Moshi, Tanzania associated with *A. fumigatus* with TR34/L98H and TR46/Y12F/T289A mutations [20]. The widespread irrational use of azole-based agricultural fungicides in the flower and horticultural industry in Kenya is a significant risk factor for azole resistance. The study aimed to determine the prevalence of triazole resistance among *Aspergillus fumigatus* from fungicide-experienced soils.

## 2. Methodology

### 2.1. Study Area

**Figure 1:**
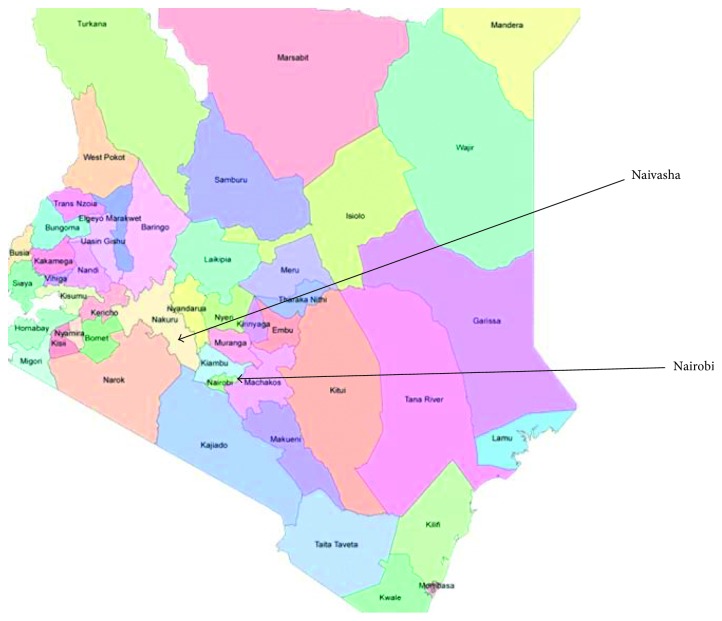
A map showing the study areas. Courtesy of www.mapsofworld.com (accessed 12 January 2015).

The study was conducted in Nairobi and Naivasha subcounty where horticultural practices and green houses are concentrated. Nairobi is the capital city of Kenya and lies at about 1°17′S and 36°49′E while Naivasha is located approximately 90 km northwest of Nairobi. It is located in Nakuru County at 0°43′S36°26E, and horticulture is the main economy. The trade names of commonly used fungicides include milraz, antracol, mistress, and victory which are broad-spectrum fungicides of ornamental, vegetable, and fruit plants (Figure 1).

### 2.2. Sampling for Environmental Isolates

A total of 156 samples were collected and analyzed. Approximately 5 g dry top surface soil from the agricultural site was collected into a sterile 15 ml Falcon tube using a sterile plastic spoon [23]. Samples were transported in a leak-proof packaging in a cool box to the Mycology Laboratory-KEMRI-Center for Microbiology Research for investigation. Clinical isolates were isolated from sputum samples of suspected aspergillosis patients and were archived at Kenya Medical Research Institute, Mycology Laboratory.

### 2.3. Fungal Culture and Identification

One gram of soil sample was mixed with 5 ml saponin, vortexed, and the debris was allowed to settle. One hundred microliters of the supernatant was transferred to 500 *µ*l of sterile normal saline and vortexed. Approximately 100 *µ*l of the suspension was cultured onto Sabouraud dextrose agar containing (a). 0.001 mg/l of itraconazole, (b) 0.001 mg/l of voriconazole, and (c) without drug (control). All the inoculated plates were incubated for 5 days at 30°C [24].

### 2.4. Broth Dilution Sensitivity Testing

*Aspergillus fumigatus* growing on azole-supplemented media were subjected to antifungal susceptibility testing against three triazoles: posaconazole (PCZ), voriconazole (VCZ), and itraconazole (ITZ) using the CLSI M38-A2 broth microdilution method [25].

### 2.5. Sequencing of CYP51A Gene

*Aspergillus fumigatus* showing high MIC against itraconazole, voriconazole, and posaconazole had their CYP51A gene sequenced for detection of mutation as previously described [26].

## 3. Results

A total 156 fungicide-exposed soil samples were analyzed, out of which 48 yielded *Aspergillus fumigatus* and 2 *A. flavus*. Antifungal susceptibility testing against three triazoles, posaconazole, voriconazole, and itraconazole, indicates that 3 isolates had MIC = 32 *µ*g/ml and other 2 had MIC = 4 *µ*g/ml against itraconazole, 5 isolates had MIC = 8 *µ*g/ml and 1 isolate had MIC = 16 *µ*g/ml against voriconazole, and 1 isolate had MIC of 32 *µ*g/ml and one had MIC = 16 *µ*g/ml against posaconazole (Table 1). Two archived clinical *Aspergillus fumigatus* from KEMRI-Mycology were used for comparison (Table 1).

Triazole resistance levels of all of the *Aspergillus fumigatus* isolates are summarized in Table 2. According to Arendrup et al. [27] breakpoints, the percentage of resistance against both itraconazole and voriconazole was 12.5% and susceptible cases at 87.5% and 85.4% against itraconazole and voriconazole, respectively. Against posaconazole, 27.1% were resistant, 60.4% were intermediates, and 12.5% were susceptible.

Three samples with high MIC were subjected to sequence analysis for the detection of mutation in CYP51A in which TR34/L98H was confirmed (Table 3).

## 4. Discussion

In our study, we report the presence of triazole-resistant *Aspergillus fumigatus* from clinical- and fungicide-experienced soils collected from Naivasha and Nairobi, Kenya. Azole-resistant *Aspergillus* spp. have been detected worldwide including Asia, Europe, Middle East, Tanzania [20], and Kenya. Kemoi et al. reported prevalence of azole-resistant *Aspergillus fumigatus* between 19.23% and 36% from both naive and experience soils. However, the study did not sequence CYP51A gene to determine the type of mutations involved [21]. The finding of this study is of great medical implications especially in African environments where resources are limited and effective treatment and early diagnosis are a challenge. Posaconazole, itraconazole, and voriconazole are the first-line drugs used in management and prevention of aspergillosis; hence, detection of environmental isolates resistant to these triazoles poses great challenge in the medical field [27, 28].

In Kenya, Naivasha subcounty is known for extensive flower farming with extensive use of fungicides. The use of azole-based fungicide in agriculture introduces antifungal pressure resulting in reduced susceptible fungi and increases azole-resistant strains [29]. Detection of *A. fumigatus* with TR34/L98H mutation is the first report in Kenya. It has been reported by several authors that isolates with mutations in TR34/L98H region have cross resistance to both medical azole and azole-based fungicides, for example, propiconazole, tebuconazole, difenoconazole, bromuconazole, and epoxiconazole [4, 12]. The TR34/L98H mutation in gene CYP51A involves the substitution of leucine 98 for histidine L98H and two 34-bp tandem copies in CYP51A gene in the promoter region [10, 30–33].

Azole-based fungicides in the environment have been linked to TR/L98H mutation in *Aspergillus fumigatus*. This form of resistance has been linked to point alteration in 220, 138, and 54 codons from patient on azole treatment [34]. Plant pathogenic molds having tandem repeat which aid in resistance against sterol demethylation inhibitor fungicides have been reported [35]. Because fungi including *A. fumigatus* and other plant fungal pathogens share the same habitat, they are constantly exposed to fungicide pressure. Therefore, if *Aspergillus* species harboring TR/L98H resistance is present in the environment, the conidia can be widely dispersed by wind and may cause infection in the susceptible individual [9].

In Moshi, Tanzania, Azole-resistant *Aspergillus fumigatus* was isolated from soil known for extensive farming. It was reported that 20% of the environmental samples harbor azole-resistant *Aspergillus fumigatus*, of which 5.5% was associated with TR46/Y122FT289A mutation and 20% TR34/L98H mutation was isolated from woody debris and soil samples [20]. The isolation of *Aspergillus fumigatus* with G54E mutation in Tanzania, Romania, and India from environmental samples is considered to occur in patients with prolonged exposure to azole [36]. The G54E mechanisms are responsible for 20.0% from India, 30.4% from Romania, and 46.4% of resistant isolates from Tanzania [36].

## 5. Conclusion

There is significant triazole resistance among environmental isolates of *Aspergillus* probably ascribed to irrational use of fungicide in agriculture and calls for legislative mechanism for control of fungicide use. This is a grave public health concern given the limited resources and the limited antifungal options available for the susceptible patients.

## Figures and Tables

**Table 1:** Sources of isolates and MIC against the three triazoles.

Number	Sources	Isolates	MIC against itraconazole	MIC against voriconazole	MIC against posaconazole
F1	Experienced soils	*Aspergillus fumigatus*	1	0.25	0.25
F2	Experienced soils	*Aspergillus fumigatus*	1	0.15	0.25
F3	Experienced soils	*Aspergillus fumigatus*	0.5	0.5	0.06
F4	Experienced soils	*Aspergillus fumigatus*	1	0.25	0.25
F5	Experienced soils	*Aspergillus fumigatus*	1	0.15	0.25
F6	Experienced soils	*Aspergillus fumigatus*	4	1	1
F7	Experienced soils	*Aspergillus fumigatus*	4	1	1
F8	Experienced soils	*Aspergillus fumigatus*	0.5	0.15	0.25
F9	Experienced soils	*Aspergillus fumigatus*	0.5	0.06	0.15
F10	Experienced soils	*Aspergillus fumigatus*	1	0.5	0.25
F11	Experienced soils	*Aspergillus fumigatus*	0.5	0.13	0.06
F12	Experienced soils	*Aspergillus fumigatus*	0.5	0.06	0.06
F13	Experienced soils	*Aspergillus fumigatus*	0.5	0.03	0.25
F14	Experienced soils	*Aspergillus fumigatus*	0.25	0.15	0.06
F15	Experienced soils	*Aspergillus fumigatus*	0.5	0.15	0.25
F16	Experienced soils	*Aspergillus fumigatus*	0.5	0.15	0.15
F17	Experienced soils	*Aspergillus fumigatus*	0.13	0.5	0.25
F18	Experienced soils	*Aspergillus fumigatus*	1	1	1
F19	Experienced soils	*Aspergillus fumigatus*	0.15	0.06	0.25
F20	Experienced soils	*Aspergillus fumigatus*	0.5	0.15	0.25
F21	Experienced soils	*Aspergillus fumigatus*	1	1	1
F22	Experienced soils	*Aspergillus fumigatus*	0.5	0.15	0.5
F23	Experienced soils	*Aspergillus fumigatus*	32	8	0.5
F24	Experienced soils	*Aspergillus fumigatus*	1	0.25	0.25
F25	Experienced soils	*Aspergillus fumigatus*	1	8	0.5
F26	Experienced soils	*Aspergillus fumigatus*	0.5	0.15	0.15
F27	Experienced soils	*Aspergillus fumigatus*	0.5	0.03	0.06
F28	Experienced soils	*Aspergillus fumigatus*	0.5	1	0.15
F29	Experienced soils	*Aspergillus fumigatus*	1	0.15	0.25
F30	Experienced soils	*Aspergillus fumigatus*	0.5	0.05	0.15
F31	Experienced soils	*Aspergillus fumigatus*	1	0.15	0.25
F32	Experienced soils	*Aspergillus fumigatus*	1	0.15	0.15
F33	Experienced soils	*Aspergillus fumigatus*	0.5	0.25	0.5
F34	Experienced soils	*Aspergillus fumigatus*	1	0.15	0.25
F35	Experienced soils	*Aspergillus fumigatus*	1	2	1
F36	Experienced soils	*Aspergillus fumigatus*	32	16	1
F37	Experienced soils	*Aspergillus fumigatus*	0.5	0.15	0.25
F38	Experienced soils	*Aspergillus fumigatus*	0.5	0.25	0.15
F39	Experienced soils	*Aspergillus fumigatus*	1	0.25	0.25
F40	Experienced soils	*Aspergillus fumigatus*	32	8	32
F41	Experienced soils	*Aspergillus fumigatus*	1	0.15	0.15
F42	Experienced soils	*Aspergillus fumigatus*	0.5	0.15	0.15
F43	Experienced soils	*Aspergillus fumigatus*	0.5	0.06	0.06
F44	Experienced soils	*Aspergillus fumigatus*	8	8	16
F45	Experienced soils	*Aspergillus fumigatus*	1	8	0.5
F46	Experienced soils	*Aspergillus fumigatus*	0.25	0.006	0.25
F47	Experienced soils	*Aspergillus fumigatus*	0.06	0.25	0.25
F48	Experienced soils	*Aspergillus fumigatus*	0.5	0.13	0.5
C21	Clinical	*Aspergillus fumigatus*	4	4	2
C40	Clinical	*Aspergillus fumigatus*	2	1	0.13

**Table 2:** Triazole resistance levels of the isolated *Aspergillus fumigatus*.

Resistance	Itraconazole (*n* (%))	Voriconazole (*n* (%))	Posaconazole (*n* (%))
Resistant	6 (12.5)	6 (12.5)	13 (27.1)
Intermediates	0	1 (2.08)	29 (60.4)
Susceptible	42 (87.5)	41 (85.4)	6 (12.5)
Total	48 (100)	48 (100)	48 (100)

**Table 3:** Sequencing analysis of CYP51A of selected *Aspergillus fumigatus*.

Number		Mutation	STRS									Sources
F36	*A. fumigatus*	TR34/L98H	14	21	8	32	9	38	8	10	18	Environmental
F40	*A. fumigatus*	TR34/L98H	14	21	8	28	9	6	8	10	18	Environmental
C21	*A. fumigatus*	TR34/L98H	14	21	8	28	9	6	8	10	18	Clinical
